# Determining the predictive capability of a Clinical Assessment Scoring Chart to differentiate severity of the clinical consequences of neonatal calf diarrhea relative to gold-standard blood gas analysis

**DOI:** 10.1371/journal.pone.0230708

**Published:** 2020-04-09

**Authors:** Patrick Dillane, Lea Krump, Emer Kennedy, Ríona G. Sayers, Gearóid P. Sayers

**Affiliations:** 1 Department of Biological and Pharmaceutical Sciences, Institute of Technology Tralee, Tralee, Co. Kerry, Ireland; 2 Animal and Grassland Research and Innovation Centre, Teagasc, Moorepark, Fermoy, Co. Cork, Ireland; Michigan State University, UNITED STATES

## Abstract

Neonatal calf diarrhea (NCD) is a major problem to calf health worldwide, in terms of both morbidity and mortality. A five-point ordinal scale clinical assessment scoring (CAS) chart was utilized to assess calves suffering from NCD-related clinical abnormalities (acidosis and dehydration) on commercial farms. The objective of this research was to determine the predictive capability of this CAS chart against gold standard blood gas parameters, designed to assist farmers in the accurate assessment of the clinical consequences of NCD. A total of 443 diarrheic and non-diarrheic calves were enrolled in the study. The CAS chart rated a calf’s health from no clinical signs to varying degrees of clinical severity on a 0 (clinically normal) to 4 (grave) scale, based on clinical indicators including calf demeanour, ear position, mobility, suckle reflex, desire-to-feed, and enophthalmos. Blood gas analysis was conducted for individual calves, consisting of pH, base excess, Na^+^, K^+^, Ca^2+^, Cl^−^, glucose, total hemoglobin, bicarbonate, anion gap, and strong ion difference. Statistical evaluation was performed by comparison of the CAS score with blood gas profiles using ordinal logistic regression and a non-parametric estimation of the Receiver Operating Characteristics (ROC). The ROC analysis indicated that the CAS chart had acceptable specificity (>95%) with low sensitivity (<60%) in differentiating clinically normal from acidotic/dehydrated cases. Assessment of individual severity classes indicated that the chart can predict and differentiate both clinically normal and advanced cases from the other severity classes (peak estimations >80%) but had reduced accuracy in differentiating mild and moderate cases (peak estimations >50%). The chart, as presented, provides a simple tool to differentiate clinically normal from calves suffering the consequences of diarrhea, but fails to accurately differentiate severity for NCD related acidosis and dehydration. Further efforts are required to enhance the sensitivity and differential diagnostic value of this type of chart.

## Introduction

Neonatal calf diarrhea (**NCD**) remains the most common source of morbidity and mortality in young bovines [1–4], characterized by a diarrhea, with accompanying acidaemia, dehydration and electrolyte imbalance [5–8]. Despite the intricate pathophysiology of NCD it is commonplace for primary producers (farmers and farm managers) to attempt diagnosis and treatment of the disease without veterinary assistance [9–12].

Blood gas analysis is widely regarded as the gold standard test to assess the degree of severity of metabolic acidosis, strong ion difference (**SID**) and electrolyte derangements in diarrheic calves [13–15]. Several studies have highlighted the strong correlation between clinical signs and blood gas variables pH, bicarbonate (**HCO**_**3**_^**-**^) and base excess (**BE**), in particular [12, 16–22]. Despite this evidence, standardization and improving the objectivity of NCD severity-based diagnosis has not been the focus of scientific research.

Scoring charts or systems have been developed as useful tools to identify health or disease status in cattle for various conditions [23–25]. By their nature, health scoring charts are based on subjective judgement, rather than objective diagnostic tests, to classify disease status [26–29]. In order to assess and enhance the accuracy of scoring systems, these charts have previously been correlated against clinical data, to associate disease status and severity with clinical scores [30, 31]. Such studies assigned values to clinical signs which were used to determine the subject’s health status. The score assigned corresponds to the animal’s risk or likelihood of disease severity [30, 31].

In an attempt to assist primary producers in the accurate assessment of the clinical consequences of NCD, primarily acidosis and dehydration, a new scoring chart (adapted from Sayers et al., 2016) was developed. The aim of this study was to determine the capability of this scoring chart to identify a calf at the preliminary stages of NCD-related clinical abnormalities and to differentiate the grade of severity relative to gold standard blood gas parameters.

## Materials and methods

### Sample population and ethical approval

A total of 443 calves (non-diarrheic: n = 393 and diarrheic: n = 50) across six farms (Farms A to F) were enrolled in the study. The sample population consisted of pooled data from previously published studies of Sayers et al. (2016) [12] (n = 82) and Dillane et al. (2018) [32] (n = 288), in addition to 73 non-diarrheic and diarrheic calves sampled for this research. The study was completed over a three-year period from 2015 to 2017. Calves ranged in age from one (>24 h) and 30 days at the time of sampling. A brief description of husbandry regimes for neonatal calves on each of the study farms is presented in S1 Table, as is the number of calves sampled on each farm. Calves were enrolled in the study, presenting with either signs of good health or varying degrees of naturally occurring diarrhea. The age, sex, and breed type (dairy or beef-cross) were recorded for each calf. The underlying cause of the condition was not identified for individual cases, as it was not the focus of the study.

Ethical approval was granted by the Health Products Regulatory Authority (HPRA) of Ireland (project number AE19132/P037) and the Teagasc Animal Ethics Committee (TAEC 81/2014). All procedures within were classified as mild, in accordance with the European Union Directive 2010/63/EU on the protection of animals used for scientific purposes.

### Generating a clinical assessment score

**Fig 1:**
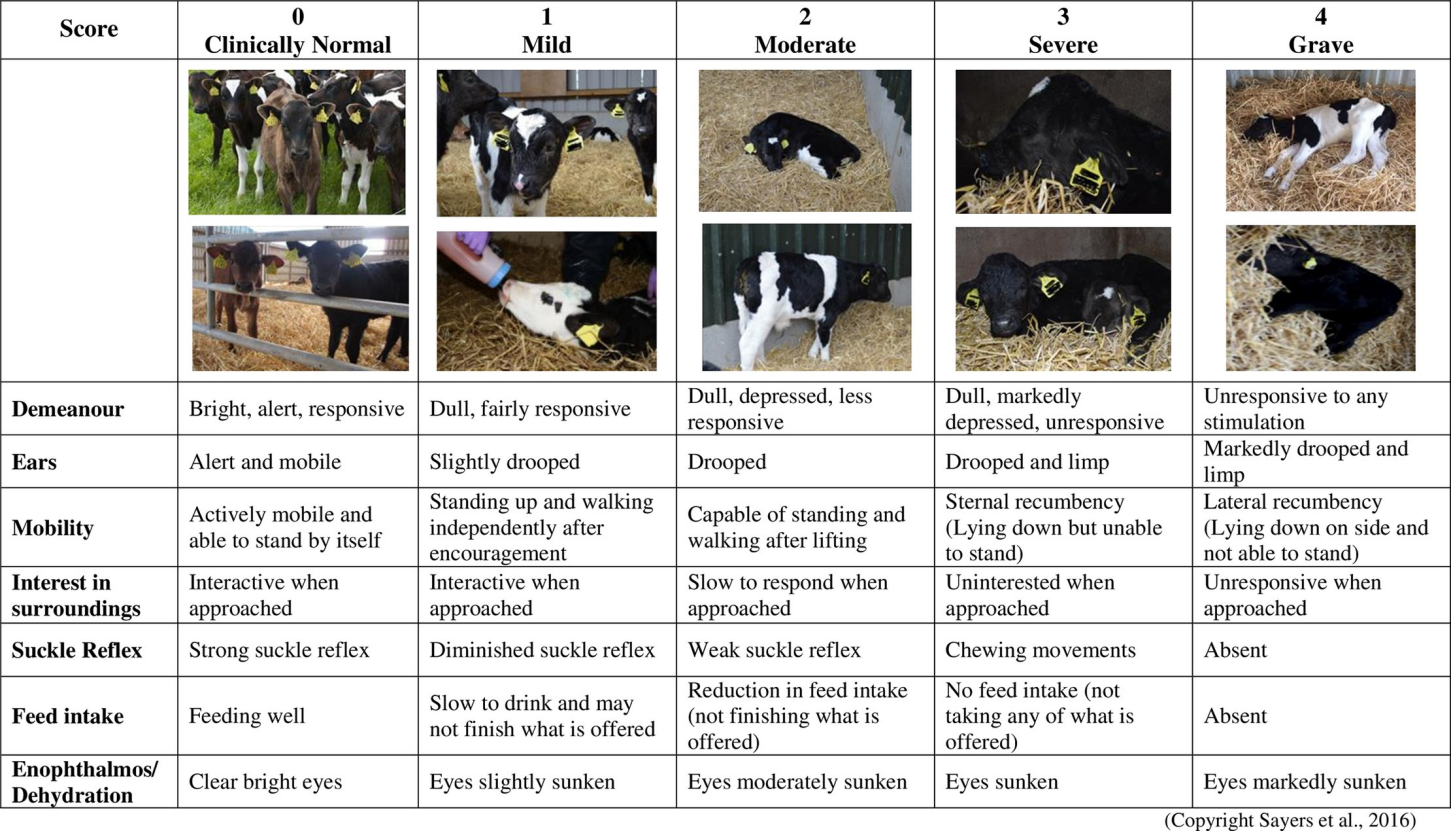
Clinical assessment scoring chart for assessment of metabolic acidosis and dehydration severity for neonatal calf diarrhea.

To evaluate a calf’s clinical status, a non-invasive five-point ordinal scale clinical assessment scoring (**CAS**) chart was developed, as presented in Fig 1 (adapted from Sayers et al., 2016). The clinical assessment was based on indicators of health, and incorporated the equal weighting of calf demeanour, ear position, mobility, interest in surroundings, suckle reflex, desire-to-feed and enophthalmos/dehydration variables. The protocol as how to ascertain a CAS score is provide in S2 Table. By way of a summary, the user identifies a score for each criterion based on the charts written and visual descriptions and generates a single digit (rounded up) average from the 7 variables. A diarrheic case was recorded if a calf’s fresh faeces had a loose or watery consistency, was malodorous and of abnormal frequency [33], and depending on the severity of the associated acidosis and dehydration were scored from 0 (clinically normal), 1 (mild), 2 (moderate), 3 (severe) and a maximum of 4 (grave). For the purpose of completeness in this study alone, the CAS chart was applied to non-diarrheic calves. The inclusion criteria of non-diarrheic calves in this study were based on the animal not being diarrheic, not having any prior recorded illness, and at the point of analysis, a clinical assessment was undertaken on each calf to evaluate its health status (all scored CAS values of 0).

All calves were assessed and scored independently but concurrently by one researcher and one veterinarian across all data and a single consensus score was recorded by agreement between the scorers at the point of assessment. All CAS scores were recorded prior to blood sampling and generation of blood gas results. Temperature was not recorded as the study instead sought to use variables that are commonly and easily observed by producers on commercial farms.

### Blood sampling

Each calf was blood sampled by jugular veni-puncture on at least one but not more than three occasions over the course of the study. A total volume of 1.5 to 2 mL of venous blood was taken into labelled heparinized 2.5 mL syringes (Cruinn Diagnostics, Dublin, Ireland). All observable air bubbles were expelled directly after sampling and the tip of each syringe was capped after sampling. Samples were gently agitated on a bottle roller for a minimum of 30 seconds following sampling to prevent formation of micro-clots. All samples were stored at room temperature and analysed within 5 minutes of sampling. A Rapidpoint 500 (Siemens, Munich, Germany) analyzer was used to test all samples using a standard temperature setting of 37°C. Blood parameters reported by the analyzer included pH, standard bicarbonate (**standard—HCO**_**3**_^**-**^) (mM), actual bicarbonate (**actual HCO**_**3**_^**-**^) (mM), partial pressure of carbon dioxide (**pCO**_**2**_) (kPa), BE (mM), Na^+^ (mM), K^+^ (mM), ionized calcium (**Ca**^**2+**^) (mM), Cl^−^ (mM), glucose (mM), total Hemoglobin (**tHb**) (g/dL), and anion gap (**AG**) (mM). The calculation of SID was based on the combined blood serum electrolyte concentrations of [Na^+^] + [K^+^]–[Cl^-^].

### Statistical analysis

Preliminary steps established the stability of the variance for each of the continuous blood gas variables based on a Shapiro-Wilks W-test, including a visual examination of ladder of powers histograms for each of the variables. In this instance, normality was confirmed for all variables investigated.

The assessment of the CAS chart as a diagnostic tool, relative to the gold standard blood gas variables pH, HCO_3_^-^, BE and was assessed using a non-parametric estimation of the Receiver Operating Characteristics (**ROC**) curve and Ordinal Logistic Regression (**OLR**). These variables were selected as they were the most revealing of the clinical consequences of diarrhea and the overall clinical picture in neonates [12, 34,35].

ROC analysis: The ROC procedures were applied to two separate examinations. The first examination determined the suitability of the CAS chart to differentiate clinically normal from NCD-associated acidotic/dehydrated cases (regardless of the illness severity). This was conducted by utilizing the lower health reference limit [32] to establish a normal/abnormal cut-off point for each gold standard blood gas variables of pH, HCO_3_^-^ and BE which were used as the dependent ‘true’ variable and plotted against the CAS score of clinically normal (CAS score of 0) or clinically abnormal (acidotic/dehydrated) (CAS score ≥1). The aim of the second ROC analysis, was to complete a theoretical determination on how close to the lower reference limit could the chart be regarded as optimal in terms of sensitivity (**Se**) and specificity (**Sp**), in differentiating acidotic/dehydrated and clinically normal calves. To achieve this, various cut-off points of the gold standard variables were determined, commencing with the lower reference limits, which was then followed by decremental unit changes from the lower reference points. At each unit change (0.01 unit change for pH and 1.0 mM unit change for HCO_3_^-^ and BE), a ROC curve was re-constructed. This stepwise process was continued until an appropriate value was identified which yielded an acceptable Se (≥ 80%) and Sp (≥ 90%) and Area Under the Curve (**AUC**) in distinguishing between clinically abnormal and normal cases [36].

OLR analysis: In order to assess the chart’s ability to differentiate the different grades of clinical abnormality, a manual backward-elimination (based on *P* > 0.30) stepwise ordinal regression procedures was utilized, followed by post-estimate predictor analysis to estimate the probability of designating a CAS value based on specified values of the significant covariate of pH, HCO_3_^-^, BE and SID. Each model, differentiated by the blood gas parameters (pH, HCO_3_^—^ std, HCO_3_^-^ - act, pCO_2_, BE, tHb, Glucose, Na^+^, K^+^, Cl^-^, Ca^2+^ AG, or SID) described the combined effect of the independent variables sex, breed type (beef or dairy—dairy breeds incorporating Holstein Friesians, Jersey cross, and Norwegian Red cross; and beef breeds incorporating Aberdeen Angus cross, Limousin cross and Belgian Blue), source farm (A, B, C, D, E, and F) and blood gas variables on the CAS value (dependent variable—CAS 3 and CAS 4 groups were pooled due to low numbers in latter group). In total, 13 models were constructed, and post-estimate predictions were conducted from the final model in each case. All data management of the results were completed using Excel (Office 2016, Microsoft Corp., Redmond, WA). Normality and statistical procedures were carried out using Stata SE v12.1. (Stata Corp LP, Texas, USA).

## Results

The descriptive statistics for the grouped CAS score cohorts are presented in Table 1, and summary statistics for age of calf enrollment is supplied in S3 Table. The ROC analysis indicated that the CAS chart, as a tool to differentiate clinically normal from NCD-related acidosis/dehydration, assessed the health status with a high degree of specificity and reduced sensitivity, for the variables investigated, as illustrated in Table 2. The results from the ROC analysis indicated that the theoretical optimal cut-off points at which the CAS chart can, with a respectable degree of accuracy, distinguish between clinically normal and acidotic/dehydrated cases are 7.34 (pH), 25.0 mM (HCO_3_^-^) and 0.6 mM (BE), as presented in Table 3 with the associated ROC curves presented in S1 Fig.

**Table 1:** Summary statistics for venous blood gas variables, differentiated by CAS score.

	CAS Score									
Blood Gas Variables	0 ^a^		1 ^b^		2 ^c^		3 ^d^		4 ^e^	
	Mean	(SEM)	Mean	(SEM)	Mean	(SEM)	Mean	(SEM)	Mean	(SEM)
pH	7.42	(0.002)	7.36	(0.012)	7.29	(0.017)	7.18	(0.040)	6.76	-
Standard—HCO_3_^-^	29.98	(0.139)	25.42	(1.020)	20.80	(0.953)	14.91	(1.546)	6.10	-
Actual -HCO_3_^-^	32.19	(0.160)	27.62	(1.176)	22.70	(1.184)	15.89	(1.540)	4.00	-
pCO_2_	5.50	(0.118)	6.55	(0.273)	6.44	(0.304)	5.72	(0.321)	-	-
Base Excess	6.61	(0.146)	1.36	(1.126)	- 4.12	(1.217)	-12.11	(2.117)	-31.60	-
Anion Gap	12.00	(0.143)	14.25	(0.676)	14.52	(1.080)	22.00	(3.064)	22.80	-
SID	44.24	(0.158)	41.86	(0.900)	37.21	(1.120)	37.89	(2.279)	26.84	-
Na^+^	137.79	(0.223)	137.22	(1.061)	132.48	(2.275)	137.64	(6.018)	135.30	-
K^+^	4.76	(0.020)	4.64	(0.141)	4.81	(0.202)	4.39	(0.301)	6.54	-
Cl^-^	98.32	(0.209)	100.00	(1.281)	100.08	(1.917)	104.14	(4.350)	115.00	-
Glucose	6.42	(0.096)	5.13	(0.181)	5.57	(0.324)	4.40	(0.522)	5.70	-
Ca^2+^	1.26	(0.003)	1.21	(0.017)	1.27	(0.023)	1.31	(0.038)	1.31	-
Total haemoglobin	11.35	(0.086)	12.86	(0.461)	13.44	(0.395)	12.86	(1.361)	19.40	-

^a^ n = 393

^b^ n = 30

^c^ n = 12

^d^ n = 7

^e^ n = 1.

**Table 2:** Non-parametric estimation of the Receiver Operating Characteristics (ROC) curve at lower reference limits of pH, HCO3-, and BE values to identify if the CAS chart can accurately differentiate clinically normal from acidotic/dehydrated calves.

Blood Gas Variable	Cut-point	Sensitivity (%)	Specificity (%)	Correctly Classified (%)	LR+	LR-	AUC
pH ^a^	7.37*	52.86	96.52	89.64	1.00	0.49	0.75
HCO_3_^-^ (mM) ^b^	28.0*	58.06	96.30	90.91	15.68	0.44	0.78
BE (mM) ^c^	2.6*	60.94	97.09	91.86	20.94	0.40	0.79

AUC = Area under the curve

^a^ clinically normal calves n = 376; acidotic/dehydrated calves n = 67.

^b^ clinically normal calves n = 378; acidotic/dehydrated calves n = 63.

^c^ clinically normal calves n = 377; acidotic/dehydrated calves n = 65.

*Lower reference range value (Dillane et al., 2018)

**Table 3:** Non-parametric estimation of the Receiver Operating Characteristics (ROC) curve at various pH, HCO3-, and BE values to identify a theoretical optimal cut-off point at which the CAS chart can accurately differentiate clinically normal from acidotic/dehydrated calves.

Cut-point	Sensitivity (%)	Specificity (%)	Correctly Classified (%)	LR+	LR-	AUC
**pH**						
7.36	71.15	96.68	93.69	21.46	0.30	0.84
7.35	76.60	96.47	94.37	21.72	0.24	0.87
**7.34**	**86.49**	**95.58**	**94.82**	**19.56**	**0.14**	**0.92**
**HCO**_**3**_^**-**^ **(mM)**						
27.0	66.67	95.41	92.27	14.52	0.35	0.81
26.0	75.00	94.31	92.73	13.17	0.27	0.86
**25.0**	**86.21**	**93.92**	**93.41**	**14.17**	**0.15**	**0.92**
**BE (mM)**						
1.6	65.38	95.90	92.31	15.93	0.36	0.81
**0.6**	**86.11**	**95.32**	**94.57**	**18.40**	**0.15**	**0.92**

Acceptable cut-points are presented in bold.

AUC = Area under the curve

**Fig 2:**
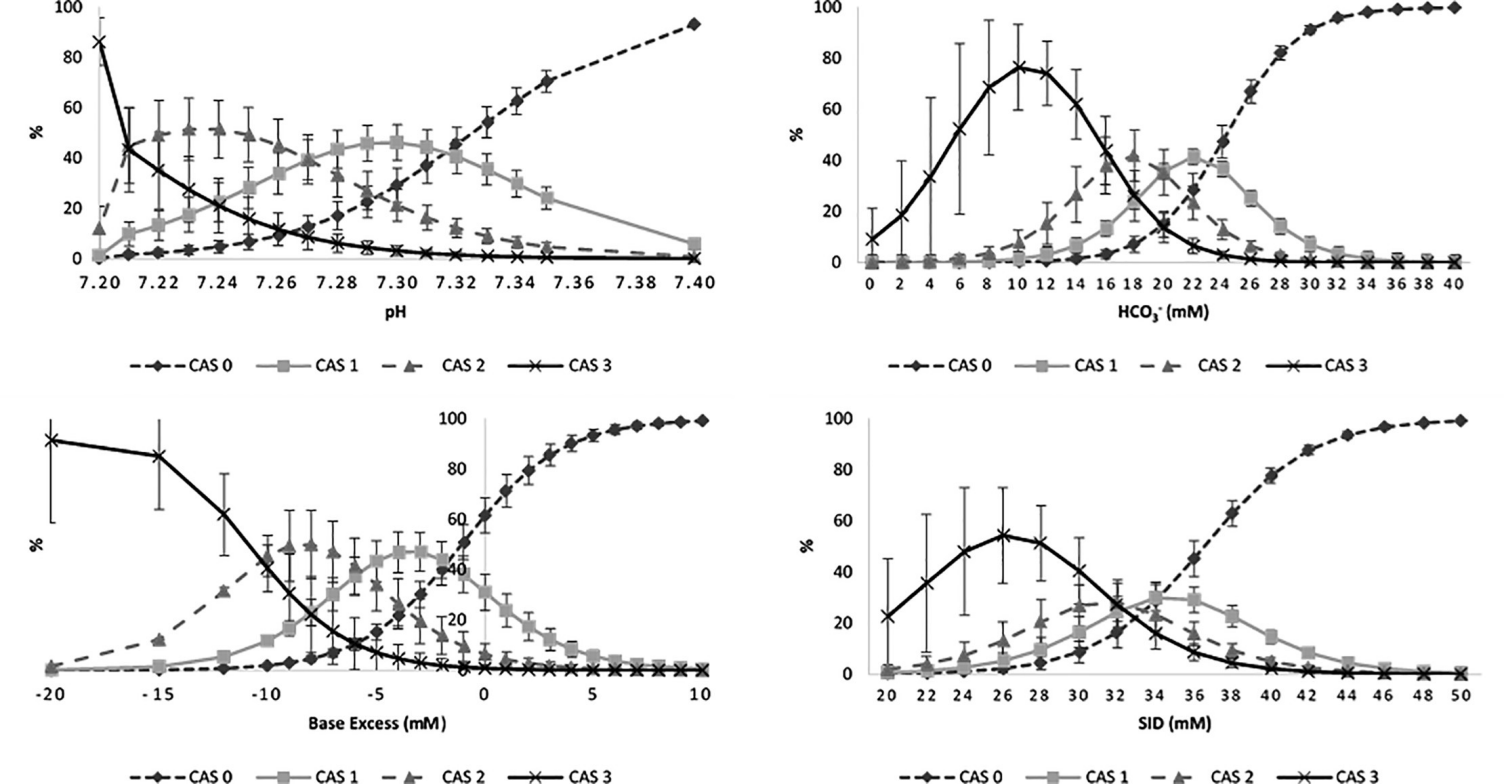
Post-estimate predictions to estimate the CAS score based on specified pH, bicarbonate, base excess, and SID blood gas values.

The results from the ORL analysis indicated that CAS score had significant associations (*P* ≤ 0.05) with 12 of the 13 blood gas variables investigated, indicating that individual unit changes of blood gas variables, with the exception of K^+^, significantly impact the CAS score. The regression analysis highlighted that the effect of sex, breed type and farm did not influence CAS score (*P* > 0.30 in all cases; presented in S4 Table). Post-estimate predictions are outlined in Fig 2. As an example, calves with a CAS score of 0 will have an 80% chance of having a pH value of 7.37 or above; calves with a CAS of 1 have a 47% chance of having a pH value of 7.30. The data demonstrated that the chart differentiates a young bovine at CAS 3 from the other CAS scores and differentiates an animal at CAS 0 from the other CAS scores, as indicated by peak estimations at greater than 80% for pH, BE and HCO_3_^-^. While individual estimation peaks can be identified for CAS 1 and CAS 2, overlap between both was evident and peak estimation was not above 50% for any of the tested variables.

## Discussion

The goal of this research was to assess the predictive capability a scoring chart that would be a useful clinical assessment tool of the clinical consequences of diarrhea in calves, for use by non-trained primary caregivers, with the specific purpose of determining the severity of acid-base and hydration status and/or to assist in monitoring recovery in response to treatment of NCD. The CAS chart assessed in this study demonstrated a satisfactory degree of accuracy in distinguishing clinically normal from acidotic/dehydrated cases and severity for calves suffering from NCD. However, the reduced sensitivity observed compared to reference limits, indicates that the chart could produce false negative results. Additionally, relative to the lower reference limit, the chart could only be regarded as theoretically optimal at 0.03 units below the lower reference limit for pH, 3 mM for HCO_3_^-^ and 2 mM for BE whereby over 92% of calves classified correctly. While this theoretical exercise holds no clinical relevance, it demonstrates a high degree of accuracy in differentiating clinical normal from acidotic and dehydrated calves, at cut-off values not too distant from the gold standard lower reference limits, its overall usefulness for a neonate that is already presenting with NCD is questionable.

Of greater importance is the charts ability to differentiate the different grades of clinical abnormality of a diarrheic calf. Assessing the chart’s ability to achieve this through ROC analysis was not possible due to inconsistent guidance in the literature about the ranges which would classify a calf into the different severity categories, and a linear relationship could not be assumed [37]. Based on post-estimate predictions, the chart was effective at differentiating both clinically normal (CAS score 0) and severely acidotic and dehydrated (CAS score 3) cases from the other classifications but had reduced accuracy in differentiating the mild and moderate classifications, where only the probability of being assigned to the correct CAS score did not pass 50%. As a chart designed to assist producers in differentiating severity of the clinical consequences of NCD, the observation that the chart does not complete this aspect with accuracy, is a limitation of its use.

Novel and inexpensive methods of quantifying the magnitude of clinical abnormalities in diarrheic calves is required [20]. The assessment of the acid-base status of a diarrheic calf is a *de facto* measure of the severity of NCD, as this attribute, in conjunction with the dehydration status, are the key determining factors in the probability of calf survival. As stated previously, blood gas profile analysis is widely considered the gold standard method for establishing the acid-base and electrolyte status of diarrheic calves [13,14, 20]. However, this approach is largely laboratory based and considered too expensive to be currently utilized on commercial farms to confirm a bovine neonate’s acid-base status or to monitor recovery [12]. A number of charts have been previously published aimed at diagnosing severity of NCD [18–21, 37]. However, difficulties relating to ease of use at pen-side application, technical terminology, and undefined boundaries of disease severity constrained their use by primary producers or untrained caregivers on commercial farms, compared to the simplified scoring system presented here.

In order to be effective, a clinical assessment score chart needs to employ simple, non-invasive, rapid and visual-based assessment techniques [29]. Additionally, the chart needs to differentiate clinically normal from acidotic/dehydrated cases, and optionally the severity of the condition, with acceptable specificity and sensitivity, proximating to relevant upper or/and lower limits of reference ranges. The categorization of disease severity has been suggested as a crucial criterion for health screening in medical and veterinary settings [22, 38,39]. Having a chart that effectively captures all the combinations and permutations of this disease, for example, a diarrheic calf that is not dehydrated yet is acidotic and recumbent, or a calf that is standing with a suckle reflex that is dehydrated, is a difficult task.

The scoring method that was applied in the chart assessed here, proposed the equal weighting of seven variables attempted to account for such variations. The meaningful post-estimate predictions identified for the pH, HCO_3_^-^, BE and SID variables indicate that the clinical variables included in the chart effectively capture the clinical state of diarrheic calves in particular and corroborate the ROC analysis results. Additionally, these same variables have been previously highlighted as closely correlated with clinical signs of NCD related acidosis and dehydration [8, 12, 16, 37, 40,41].

Further enhancements to improve disease severity classification for this chart is required. While the current system was designed to be a visual and non-invasive evaluation of the condition, the adoption of weighted variables and the inclusion of clinical data such as rectal temperature, measurement of skin tenting and measure of palpebral reflex to associate disease severity with clinical scores as a means of ‘assessing’ a chart system would likely lead to a more standardized approach with reduced bias [23, 29–31]. Furthermore, some of the clinical criteria (calf demeanour, ear position and enophthalmos) are subjective in nature, increasing the likelihood of variability in scores between users. While a pictorial-based approach reduces this subjectivity, and the averaging of results dilutes its impact, this variability should be considered, particularly if weighting is to be applied to specific criteria.

A benefit of previous scoring systems for veterinarians [18–21, 37] was establishing a link between the status of the calf and the degree of dehydration and or acidosis, thereby informing on the nature and level of treatment required. Such a link was not applied to this chart as it was aimed at primary producers. However, the ability of the caregiver to associate an electrolyte to a given severity classification and determine a treatment protocol tailored to the animal’s need or aid in their decision to call a veterinarian in more advanced cases would represent another area of potential improvement for scoring systems of this nature [4, 10–12]. Regardless, as there have been several publications describing protocols to address NCD cases, from mild/moderate [10, 12, 33] to more advanced cases [16–18], a chart that enables the caregiver to make this differentiation of severity without the need for specialized diagnostic equipment is likely to be of benefit.

## Conclusions

The results of the current study indicate that the use of a CAS chart, when coupled with simplified written descriptions and pictographic references, as presented here, is an economical assistant to caregivers to differentiate clinically normal from abnormal cases but fails to accurately differentiate the severity of metabolic acidosis and dehydration of diarrheic calves for on-farm conditions. Further efforts are required to enhance the sensitivity and differential diagnostic value of this type of chart.

## Supporting information

S1 Table Description of calf husbandry regimes on each study farm for neonatal calves. (PDF)

S2 Table Clinical assessment score protocol. (PDF)

S3 Table Descriptive statistics for calf age at study enrolment for each CAS score cohort. (PDF)

S4 Table Manual backward elimination stepwise ordinal regression procedure between CAS score and blood gas variables. (PDF)

S1 Fig Receiver Operating Characteristics (ROC) curves at theoretical optimal values for pH, HCO3-, and BE values. (PDF)
