# Supplementary material for: Determining the predictive capability of a Clinical Assessment Scoring Chart to differentiate severity of the clinical consequences of neonatal calf diarrhea relative to gold-standard blood gas analysis
Source: PLoS One. 2020 Apr 9;15(4):e0230708. doi: 10.1371/journal.pone.0230708 (PMC7144965; doi:10.1371/journal.pone.0230708)
Supplement: S1 Table — (PDF) [file pone.0230708.s001.pdf]

S1 Table. Description of calf husbandry regimes on each study farm for neonatal calves.

Farm[1]	Breeds[2]	Housing Management	Milk feeding system
A[3]	HF, JeX, AAX, LMX, NRX	Males and females housed separately. Individual calf pen followed by group pens (up to 20 animals) at 3 d of age. Deep straw bedding in all pens. Ad libitum water and creep feed available.	Automatic feeders with an allowance of 6 L of milk replacer per calf per day as a routine.
B[4]	HF, JeX, NRX	Males and females housed separately. Individual calf pen followed by group pens (up to 25 animals) at 3 d of age. Deep straw bedding in all pens. Ad libitum water and creep feed available.	Manual multi-calf feeding buckets with an allowance of 6 L of milk replacer or whole milk per calf per day.
C[5]	HF, JeX, AAX	Males and females housed separately. Individual calf pen followed by group pens (up to 12 animals) at 3 d of age. Deep straw bedding in all pens. Ad libitum water and creep feed available.	Automatic feeders with an allowance of 6 L of milk replacer per calf per day as a routine.
D[6]	HF, JeX, BB	Individual calf pen followed by group pens (up to 15 animals) at 2 d of age. Deep straw bedding in all pens. Ad libitum water and creep feed available.	Manual multi-calf feeding buckets with an allowance of 6 L of milk replacer or whole milk per calf per day.
E[7]	HF, JeX	Deep straw-bedded group pens from birth (up to 20 animals). Ad libitum water and creep feed available.	Manual multi-calf feeding buckets with an allowance of 6 L of milk replacer per calf per day.
F[8]	HF, JeX	Straw-bedded group pens from birth (up to 10 animals), moving to woodchip bedded group pens (up to 20 animals) from approximately 2 wk of age. Ad libitum water and creep feed available.	Manual multi-calf feeding buckets with an allowance of 4 L of milk replacer per calf per day.

[1] Farms A, B and C are research farms, and D, E and F are commercial dairy farms.
[2] HF = Holstein-Friesian; JeX = Jersey cross; NRX = Norwegian Red cross; AAX = Aberdeen Angus cross; LMX = Limousin cross; BB = Belgian Blue.
[3] n = 106; [4] n = 132; [5] n = 128; [6] n = 46; [7] n = 7; [8] n = 24.
