# Supplementary material for: Determining the predictive capability of a Clinical Assessment Scoring Chart to differentiate severity of the clinical consequences of neonatal calf diarrhea relative to gold-standard blood gas analysis
Source: PLoS One. 2020 Apr 9;15(4):e0230708. doi: 10.1371/journal.pone.0230708 (PMC7144965; doi:10.1371/journal.pone.0230708)
Supplement: S2 Table — (PDF) [file pone.0230708.s002.pdf]

S2 Table. Clinical assessment score protocol.

Calves that present with diarrhoea, evidenced by faeces with a loose or watery consistency, malodorous and/or abnormal frequency, are assessed using the chart as follows:

1. The user visually examines the calf for each of the seven indicators of health - calf demeanour, ear position, mobility, interest in surroundings, suckle reflex, desire-to-feed and enophthalmos/dehydration – based on the written and visual description provided in the chart.
2. For each indicator, a score is recorded on a scale of 0 to 4.
3. The subtotal scores are summated and averaged to provide the calf with a single digit (rounded up) CAS score on a five point scale of healthy (0) to severe (4).
