# Supplementary material for: Determining the predictive capability of a Clinical Assessment Scoring Chart to differentiate severity of the clinical consequences of neonatal calf diarrhea relative to gold-standard blood gas analysis
Source: PLoS One. 2020 Apr 9;15(4):e0230708. doi: 10.1371/journal.pone.0230708 (PMC7144965; doi:10.1371/journal.pone.0230708)
Supplement: S3 Table — (PDF) [file pone.0230708.s003.pdf]

**S3 Table. Descriptive statistics for calf age at study enrolment for each CAS score cohort.**

| CAS Score | Obs. | Mean (Days) | SD | Range (Days) |
|---|---|---|---|---|
| CAS 0 | 393 | 11.8 | 9.68 | 1,30 |
| CAS 1 | 30 | 18.32 | 4.42 | 13,28 |
| CAS 2 | 12 | 19.8 | 3.49 | 16,23 |
| CAS 3 | 7 | 15.4 | 2.88 | 11,18 |
| CAS 4 | 1 | - | - | - |

Obs. = Observations; SD = Standard Deviation
