# Supplementary material for: Determining the predictive capability of a Clinical Assessment Scoring Chart to differentiate severity of the clinical consequences of neonatal calf diarrhea relative to gold-standard blood gas analysis
Source: PLoS One. 2020 Apr 9;15(4):e0230708. doi: 10.1371/journal.pone.0230708 (PMC7144965; doi:10.1371/journal.pone.0230708)
Supplement: S4 Table — (PDF) [file pone.0230708.s004.pdf]

S4 Table. Manual backward elimination stepwise ordinal regression procedure between CAS score and blood gas variables.

	CAS Score		
	**Odds Ratio**	**SEM**	**P Value**
pH	< 0.001	< 0.001	0.001
Standard - HCO_3^-	0.606	0.031	0.001
Actual - HCO_3^-	0.665	0.028	0.001
pCO_2	1.157	0.078	0.031
BE	0.646	0.030	0.001
AG	1.350	0.057	0.001
SID	0.698	0.032	0.001
Na^+	0.885	0.037	0.004
K^+	-	-	-
Cl^-	1.095	0.028	0.001
Glucose	0.477	0.070	0.001
Ca^{2+}	0.009	0.020	0.033
tHb	1.815	0.175	0.001

'- 'indicates variable eliminated from the model construct ($P > 0.30$)
