# Supplementary material for: Determining the predictive capability of a Clinical Assessment Scoring Chart to differentiate severity of the clinical consequences of neonatal calf diarrhea relative to gold-standard blood gas analysis
Source: PLoS One. 2020 Apr 9;15(4):e0230708. doi: 10.1371/journal.pone.0230708 (PMC7144965; doi:10.1371/journal.pone.0230708)
Supplement: S1 Fig — (PDF) [file pone.0230708.s005.pdf]

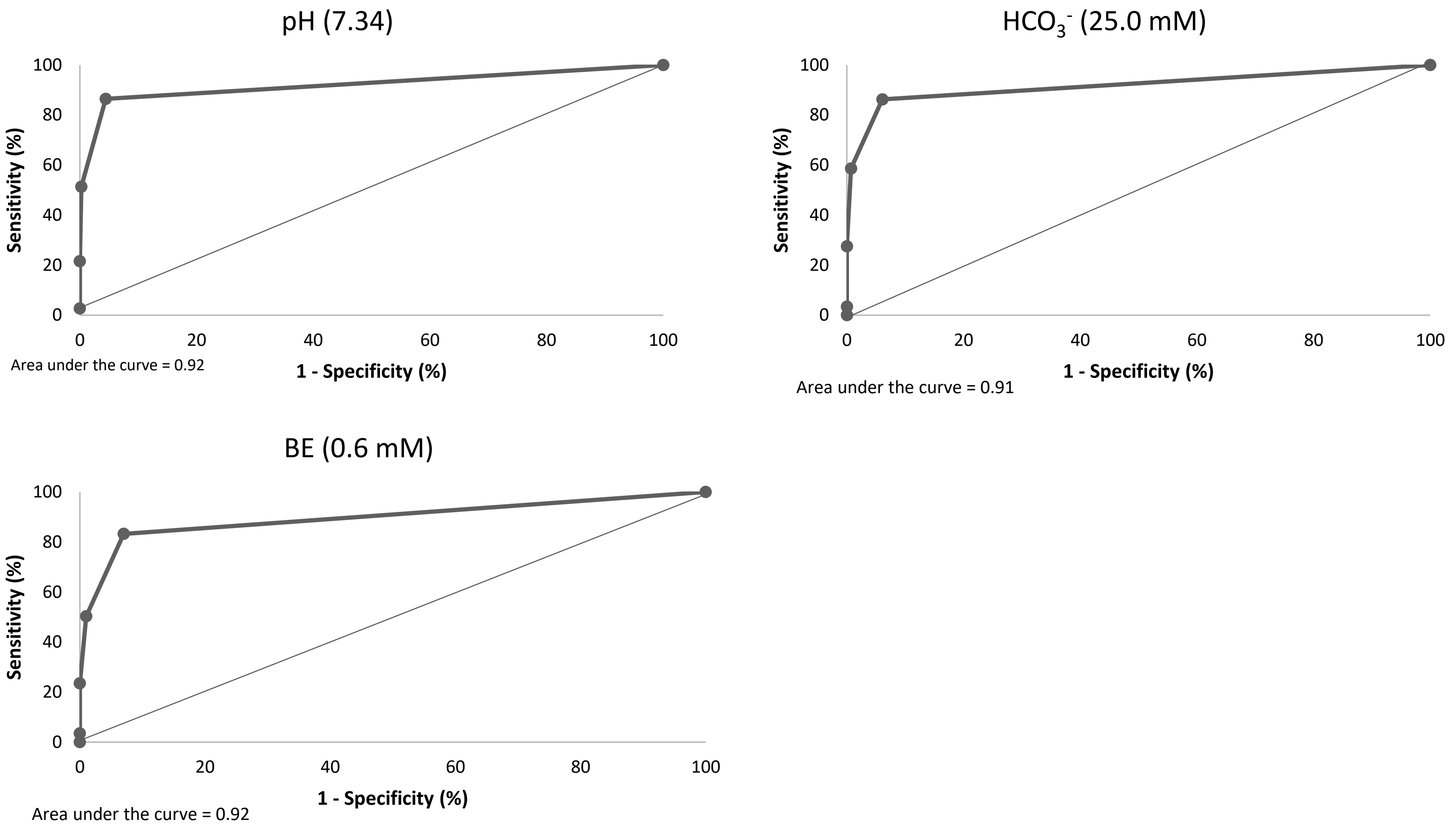

S1 Fig. Receiver operating characteristics (ROC) curves at theoretical optimal values for pH, HCO_3^-, and BE values.
